# Supplementary material for: On the relationship between tumour growth rate and survival in non-small cell lung cancer
Source: PeerJ. 2017 Nov 29;5:e4111. doi: 10.7717/peerj.4111 (PMC5712205; doi:10.7717/peerj.4111)
Supplement: Supplemental Information 1 [file peerj-05-4111-s001.docx]

**Supplementary Information**

This document contains the diagnostic plots and tables of parameter values for the SLD time-series analysis conducted using the model described in the main paper.

**Erlotinib – Forwards Alignment**


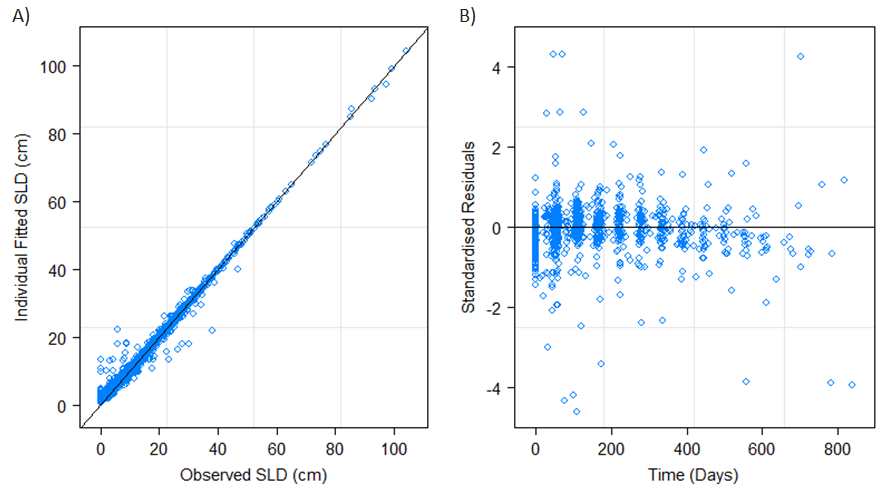


Figure S1. Panel A shows the observed SLD values against the individual fitted. Panel B shows the individual standardised residuals over time.

Table S1. Provides estimated mean and standard deviation (S.D.) values together with approximate 95% confidence intervals for the parameter distributions of the model described in the main paper. Estimate of the within group standard error (S.E.) of the residuals with 95% CI is also provided.

| log(A) | | log(B) | | log(C) | | Residual |
| --- | --- | --- | --- | --- | --- | --- |
| Mean  (95% C.I.) | S.D.  (95% C.I.) | Mean  (95% C.I.) | S.D.  (95% C.I.) | Mean  (95% C.I.) | S.D.  (95% C.I.) | S.E.  (95% C.I.) |
| 2.79  (2.72, 2.85) | 0.62  (0.57, 0.67) | -5.04  (-5.22,  -4.85) | 1.09  (0.94, 1.26) | -5.60  (-5.72,  -5.48) | 0.89  (0.80, 0.97) | 2.35  (2.22, 2.49) |

**Erlotinib – Reverse Alignment**


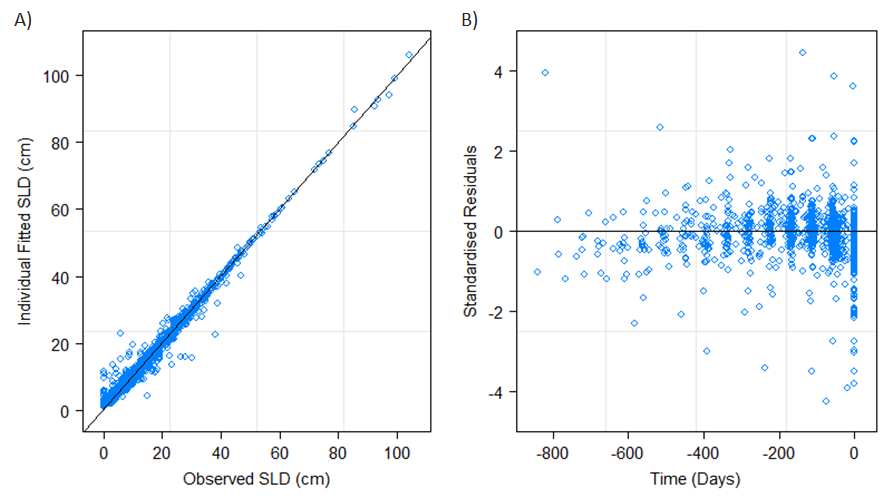


Figure S2. Panel A shows the observed SLD values against the individual fitted. Panel B shows the individual standardised residuals over time.

Table S2. Provides estimated mean and standard deviation (S.D.) values together with approximate 95% confidence intervals for the parameter distributions of the model described in the main paper. Estimate of the within group standard error (S.E.) of the residuals with 95% CI is also provided.

| log(A) | | log(B) | | log(C) | | Residual |
| --- | --- | --- | --- | --- | --- | --- |
| Mean  (95% C.I.) | S.D.  (95% C.I.) | Mean  (95% C.I.) | S.D.  (95% C.I.) | Mean  (95% C.I.) | S.D.  (95% C.I.) | S.E.  (95% C.I.) |
| 2.80  (2.72, 2.87) | 0.71  (0.66, 0.77) | -4.83  (-4.94,  -4.72) | 0.50  (0.42, 0.60) | -5.74  (-5.87,  -5.61) | 0.91  (0.83, 1.00) | 2.75  (2.56, 2.95) |

**Docetaxel – Forwards Alignment**


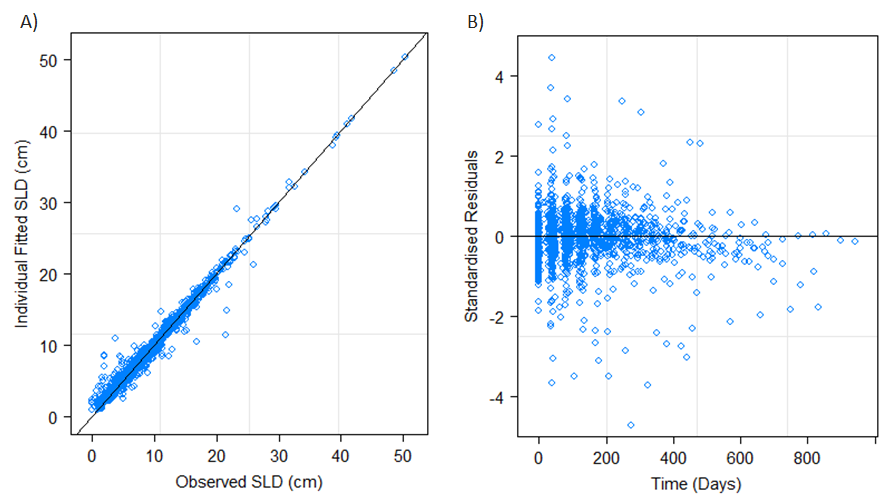


Figure S3. Panel A shows the observed SLD values against the individual fitted. Panel B shows the individual standardised residuals over time.

Table S3. Provides estimated mean and standard deviation (S.D.) values together with approximate 95% confidence intervals for the parameter distributions of the model described in the main paper. Estimate of the within group standard error (S.E.) of the residuals with 95% CI is also provided.

| log(A) | | log(B) | | log(C) | | Residual |
| --- | --- | --- | --- | --- | --- | --- |
| Mean  (95% C.I.) | S.D.  (95% C.I.) | Mean  (95% C.I.) | S.D.  (95% C.I.) | Mean  (95% C.I.) | S.D.  (95% C.I.) | S.D.  (95% C.I.) |
| 2.09  (2.03, 2.15) | 0.59  (0.55, 0.64) | -5.27  (-5.38,  -5.16) | 0.59  (0.50, 0.69) | -5.97  (-6.07,  -5.86) | 0.80  (0.72, 0.89) | 1.06  (1.00, 1.11) |

**Docetaxel – Reverse Alignment**


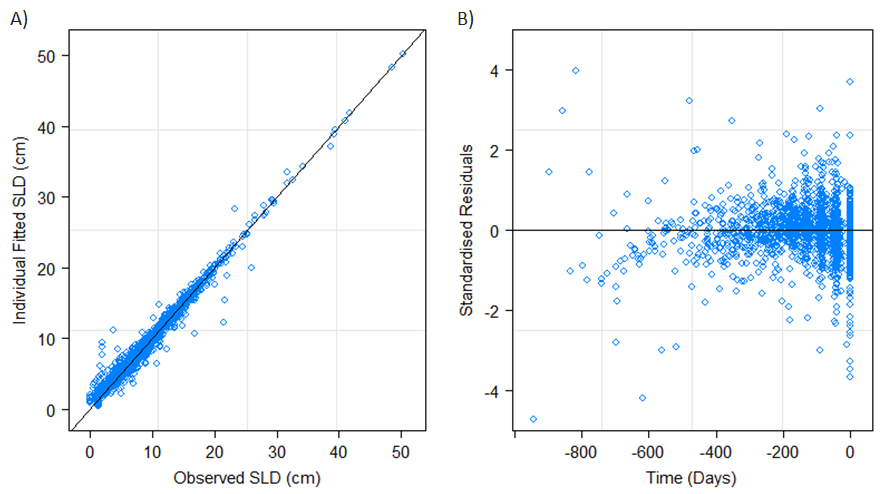


Figure S4. Panel A shows the observed SLD values against the individual fitted. Panel B shows the individual standardised residuals over time.

Table S4. Provides estimated mean and standard deviation (S.D.) values together with approximate 95% confidence intervals for the parameter distributions of the model described in the main paper. Estimate of the within group standard error (S.E.) of the residuals with 95% CI is also provided.

| log(A) | | log(B) | | log(C) | | Residual |
| --- | --- | --- | --- | --- | --- | --- |
| Mean  (95% C.I.) | S.D.  (95% C.I.) | Mean  (95% C.I.) | S.D.  (95% C.I.) | Mean  (95% C.I.) | S.D.  (95% C.I.) | S.D.  (95% C.I.) |
| 2.04  (1.98, 2.11) | 0.67  (0.62, 0.72) | -5.17  (-5.26,  -5.08) | 0.47  (0.40, 0.56) | -5.95  (-6.04,  -5.87) | 0.55  (0.50, 0.61) | 1.06  (1.02, 1.12) |

**Paclitaxel/Carboplatin – Forwards Alignment**


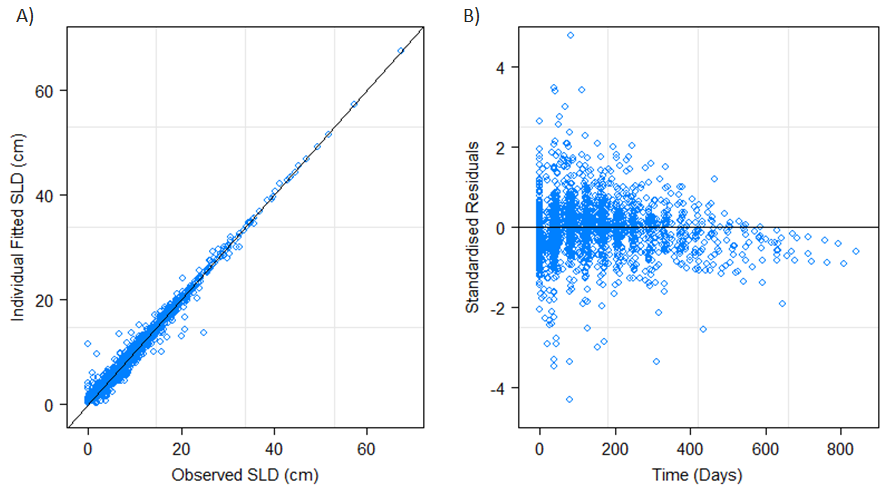


Figure S5. Panel A shows the observed SLD values against the individual fitted. Panel B shows the individual standardised residuals over time.

Table S5. Provides estimated mean and standard deviation (S.D.) values together with approximate 95% confidence intervals for the parameter distributions of the model described in the main paper. Estimate of the within group standard error (S.E.) of the residuals with 95% CI is also provided.

| log(A) | | log(B) | | log(C) | | Residual |
| --- | --- | --- | --- | --- | --- | --- |
| Mean  (95% C.I.) | S.D.  (95% C.I.) | Mean  (95% C.I.) | S.D.  (95% C.I.) | Mean  (95% C.I.) | S.D.  (95% C.I.) | S.D.  (95% C.I.) |
| 2.40  (2.34, 2.47) | 0.68  (0.63, 0.73) | -4.51  (-4.61,  -4.42) | 0.85  (0.78, 0.93) | -6.27  (-6.36,  -6.18) | 0.76  (0.69, 0.84) | 1.21  (1.16, 1.26) |

**Paclitaxel/Carboplatin – Reverse Alignment**


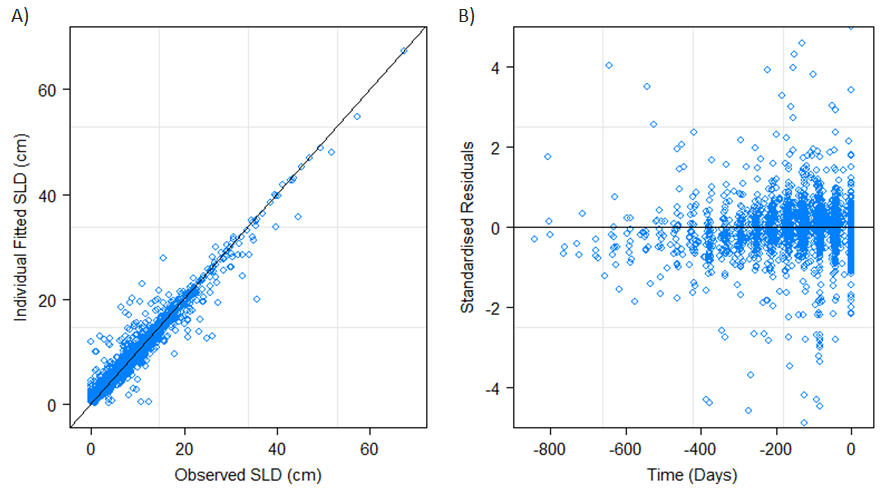


Figure S6. Panel A shows the observed SLD values against the individual fitted. Panel B shows the individual standardised residuals over time.

Table S6. Provides estimated mean and standard deviation (S.D.) values together with approximate 95% confidence intervals for the parameter distributions of the model described in the main paper. Estimate of the within group standard error (S.E.) of the residuals with 95% CI is also provided.

| log(A) | | log(B) | | log(C) | | Residual |
| --- | --- | --- | --- | --- | --- | --- |
| Mean  (95% C.I.) | S.D.  (95% C.I.) | Mean  (95% C.I.) | S.D.  (95% C.I.) | Mean  (95% C.I.) | S.D.  (95% C.I.) | S.D.  (95% C.I.) |
| 1.94  (1.86, 2.02) | 0.79  (0.73, 0.86) | -4.65  (-4.76,  -4.54) | 0.51  (0.40, 0.65) | -5.33  (-5.41,  -5.26) | 0.67  (0.62, 0.73) | 2.07  (1.99, 2.16) |
